# Modelling host–pathogen interactions: Galleria mellonella as a platform to study Pseudomonas aeruginosa response to host-imposed zinc starvation

**DOI:** 10.1099/mic.0.001524

**Published:** 2025-01-22

**Authors:** Emma Michetti, Tulasi Abinya Mandava, Valerio Secli, Francesca Pacello, Andrea Battistoni, Serena Ammendola

**Affiliations:** 1 Department of Biology, Tor Vergata University of Rome, Rome, Italy

**Keywords:** *amiA*, *in vivo *model, nutritional immunity, zinc transporters, Zur-regulated operons

## Abstract

Nutritional immunity, a key component of the vertebrate innate immune response, involves the modulation of zinc availability to limit the growth of pathogens. *Pseudomonas aeruginosa* counteracts host-imposed zinc starvation through metabolic adaptations, including reprogramming of gene expression and activating efficient metal uptake systems. To unravel how zinc shortage contributes to the complexity of bacterial adaptation to the host environment, it is critical to use model systems that mimic fundamental features of *P. aeruginosa*-related diseases in humans. Among available animal models, *Galleria mellonella* has recently emerged as a promising alternative to mammalian hosts. This study aims to evaluate whether *G. mellonella* can recapitulate the zinc-related nutritional immunity responses observed in mammalian infections. Our results show that, upon *P. aeruginosa* infection, the larvae upregulate several zinc transporters, suggesting an active redistribution of the metal in response to the pathogen. Additionally, *P. aeruginosa* colonizing the larvae induces Zn uptake regulator-controlled genes, consistent with bacterial adaptation to zinc starvation. Disruption of bacterial zinc uptake capability significantly reduces *P. aeruginosa* virulence, underscoring the importance of zinc acquisition in pathogenesis also within this model host. As a proof of concept, we also demonstrate that this *in vivo* model can serve as a viable preliminary screening tool to unveil novel players involved in *P. aeruginosa* response to zinc starvation, offering valuable insights into the host–pathogen battle for micronutrients.

## Introduction

The innate immune response is the body’s first line of defence against infections, providing immediate protection against pathogens. Among the strategies of the innate immune response, nutritional immunity involves the manipulation of the concentration of essential nutrients to control the growth and virulence of invading micro-organisms [1,2]. Metals, such as iron (Fe), zinc (Zn), manganese (Mn) and copper (Cu), are essential micronutrients due to their roles in many cellular functions [3]. On the other hand, these metals can be toxic if they accumulate over a certain threshold, triggering potentially harmful redox reactions and protein mismetallation [4]. Nutritional immunity exploits both the essentiality and toxicity of these metals, manipulating their concentration to starve or poison the pathogens [5,7]. The latter have evolved numerous strategies to evade such host defences, including highly effective uptake pathways to ensure an adequate metal supply in case of stringency and efflux system to prevent intracellular toxic metal accumulation [8].

*Pseudomonas aeruginosa* is an opportunistic Gram-negative pathogen responsible for acute and chronic infections in immunocompromised individuals. It is one of the leading causes of life-threatening lung infections in cystic fibrosis (CF) patients. Due to the continuous emergence of antimicrobial resistance traits, *P. aeruginosa* is included in the priority list of pathogens against which urgent research for new active compounds is required [9]. Moreover, a threatening aspect of *P. aeruginosa* is its ability to colonize diverse environments and face nutrient fluctuations thanks to its notable metabolic plasticity [10,11]. Adequate availability of Zn influences the expression of *P. aeruginosa* virulence traits, including alginate production, biofilm formation, protease secretion, siderophore biosynthesis and motility, essential for establishing lethal infections [12,14].

The capacity of *P. aeruginosa* to thrive in Zn-limited environments, such as the human lungs [12,15], largely depends on the upregulation of multiple Zn acquisition systems, which are transcriptionally controlled by the Zn uptake regulator (Zur). Among these systems, *znuABC* is extensively studied and conserved among pathogens and encodes for an ATP-dependent ABC transporter, facilitating the high-affinity uptake of free Zn ions from the periplasm to the intracellular environment [16]. Additionally, a mechanism to counteract Zn starvation involves synthesizing and releasing the metallophore pseudopaline encoded by the *zrmABCD* operon. Pseudopaline binds Zn extracellularly, capturing it from host-released molecules such as calprotectin, and imports it back to the cytoplasm [17,18]. These genes exhibited significant overexpression in *P. aeruginosa* isolates obtained from infected tissues, emphasizing their pivotal role in colonizing the host environment [19].

Conversely, *P. aeruginosa* activates mechanisms to export excess metals when Zn levels exceed a certain threshold. One notable system for metal efflux is encoded by the *czcABCD* operon, which belongs to the resistance-nodulation-division group of the heavy metal efflux family. This system can expel excess Zn, cadmium (Cd) and cobalt (Co) directly outside the cell from the cytoplasmic or periplasmic compartments [20]. Induction of *czcCBA* in *P. aeruginosa* has been observed upon phagocytosis by macrophages, suggesting a response to a toxic metal boost within the phagolysosome to combat microbial intracellular survival [21].

The strategies of *P. aeruginosa* to face host nutritional immunity represent a promising target for developing innovative antimicrobial therapies. Many studies have been conducted *in vitro* or mammalian models to exploit the possibility of hampering microbial growth by interfering with metal homeostasis [22,25]. However, ethical and financial issues related to using mammals as models increased the interest in exploring alternative organisms, such as zebrafish, insects and nematodes, which recapitulate human nutritional immunity strategies to some extent [26,28].

*Galleria mellonella* (greater wax moth) has emerged as a valuable model for preliminary studies in microbial pathogenesis and the development of antimicrobial compounds [29]. It offers several advantages, including the ability to thrive at temperatures similar to the human body (37 °C), which is advantageous for studying human pathogens that require this temperature for optimal growth. Moreover, it possesses an innate immune system that exhibits remarkable similarities to that of mammals, including physical barriers and cellular and humoral defences [30]. It is unknown, however, whether the response of *G. mellonella* to human pathogens involves nutritional immunity mechanisms, such as the modulation of Zn availability.

This work aimed to investigate whether *G. mellonella* is a suitable model for studying *P. aeruginosa* infections, particularly in mimicking the Zn availability condition encountered by this pathogen in the human lungs. Our results reveal that infected larvae upregulate genes involved in Zn transport, strongly suggesting the activation of a nutritional immune strategy to combat pathogens. We demonstrated that *P. aeruginosa* colonizing *G. mellonella* experiences Zn stringency and that bacterial Zn uptake systems are crucial for its pathogenicity. These findings support the use of *G. mellonella* as a model organism to identify novel players in the competition for micronutrients between the host and the pathogen.

## Methods

### Bacterial strains and growth conditions

Bacterial strains used in this work are listed in Table S1 (available in the online Supplementary Material) and were plated on *Pseudomonas* Isolation Agar (PIA) (Becton Dickinson) or Luria–Bertani (LB) agar (tryptone 10 g l^−1^, yeast extract 5 g l^−1^ and NaCl 10 g l^−1^) supplemented with antibiotic when needed (for *Escherichia coli*, gentamicin 10 μg ml^−1^ and kanamycin 50 μg ml^−1^; for *P. aeruginosa* gentamicin 100 μg ml^−1^) and incubated at 37 °C. Liquid cultures were routinely grown in LB at 37 °C under shaking. Vogel-Bonner Minimal Medium (MgSO_4_–7H_2_O 0.192 g l^−1^, citric acid 2 g l^−1^, anhydrous K_2_HPO_4_ 10 g l^−1^, NaNH_4_HPO_4_ –4H_2_O 3.5 g l^−1^ and glucose 2 g l^−1^) supplemented with EDTA 5 µm (E-VBMM) was used for bacterial growth under Zn limiting conditions, as already described [17].

### Construction of promoter-lux fusions

Fragments spanning ~200–300 bp upstream of the coding sequences of *rpsL*, *zrmA* and *czcA* were amplified from *P. aeruginosa* PA14 genomic DNA (extracted with Quick-DNA Fungal/Bacterial Kit, Zymo Research) by PCR, with Expand^TM^ High-Fidelity DNA Polymerase (Roche Life Science) and primers listed in Table S2. Each fragment was digested with EcoRI and SacI restriction enzymes (Thermo Fisher Scientific), purified from agarose gel using the Zymoclean Gel DNA Recovery Kit (Zymo Research) and ligated into the pETSlux plasmid [31] using the T4 Ligase (New England Biolabs). *E. coli* DH5α chemical competent cells were transformed with the ligation mixtures, and the plasmids were then isolated from gentamicin-resistant clones using the Plasmid Isolation Kit (Zymo Research). The insertion of promoters was verified by EcoRI/SacI restriction. Plasmids with the promoters-*lux* fusion (Table S1) were transferred to the *P. aeruginosa* PA14 wt and *znuAzrmB::FLP* mutant strains by triparental mating, using HB101 pRK2013 as the helper strain. Exconjugants were selected on PIA supplemented with gentamicin 100 mg l^−1^.

### *P. aeruginosa* growth and luminescence analyses

Overnight LB cultures of *P. aeruginos*a strains carrying P*zrmA*-lux, P*czcA*-lux or P*rpsL*-lux plasmids were diluted 1:1000 in E-VBMM supplemented or not with different amounts of ZnSO_4_. Bacterial growth and luminescence were monitored in a black microplate with a transparent bottom by a SUNRISE microtiter-plate reader (Tecan), simultaneously recording the OD at 595 nm (OD_595_) and the relative luminescence units (RLUs) (integration time 1000 ms) over time.

### *P. aeruginosa amiA* mutant construction

The *amiA* deletion mutant was obtained using the gene replacement method [32] with minor modifications [13]. The gentamicin resistance cassette was obtained by BamHI (Thermo Fisher Scientific) digestion of plasmid pSP856 (Table S1). The 5′ and the 3′ terminal fragments of *amiA* were amplified using PA14 DNA and the primers listed in Table S2 and digested with EcoRI/BamHI and BamHI/HindIII, respectively. Cloning of the 5′ and 3′ fragments and the gentamicin resistance cassette in plasmid pEX18Tc and mobilization of the resulting plasmid to PA14 wt by tri-parental mating were performed as already described [13]. The *amiA* deletion in PA14 was confirmed by PCR using the primers in Table S2.

### Injection of *G. mellonella* larvae and time-to-death evaluation

*G. mellonella* last-instar larvae were obtained from a local vendor and used within the same day for the experiments. *P. aeruginosa* overnight cultures were diluted 1 : 20 in LB broth and grown until an OD at 600 nm (OD_600_) of 0.6–0.8. Bacteria were then diluted to ~2500 c.f.u. ml^−1^ in sterile PBS, pH 6.4. Groups of 5–15 larvae, weighing ~200 mg each larva, were surface sterilized with a cotton swab dipped in 70% ethanol before injection. Ten microlitres of the bacterial suspension were injected into the larval hemolymph through the last left proleg using a Hamilton microsyringe equipped with a 30-gauge needle (Hamilton Company). In each experiment, a group of six to ten PBS (pH 6,4)-injected larvae (mock) was also included to ensure that the death of the larvae was not due to needle injury. Suitable aliquots of the suspension used for injection were plated on PIA plates for counting to verify the bacterial infecting dose. After the injection, the larvae were placed into Petri dishes and kept at 37 °C in the darkness. For time-to-death experiments, the death of the larvae was assessed according to symptoms such as the lack of movement, no reactivity to stimuli and complete melanization [33].

### *P. aeruginosa* viability and competition assays in *G. mellonella*

The number of viable bacteria from the hemolymph of infected larvae was evaluated at 14, 18, 24 and 40 h post-infection (hpi). The abdomen of each larva was surface sterilized with a cotton swab dipped in 70% ethanol and pricked with a sterile needle to collect 10 µl of hemolymph. Serial dilutions of the hemolymph were plated on PIA and incubated at 37 °C for 18 h for colony counting.

For competition assays, *P. aeruginosa* PA14 wt and mutant strains grown overnight in LB were diluted 1:20 in fresh medium and let grow until they reached OD_600_ of 0.6–0.8. Bacteria were diluted in PBS, pH 6.5, at 2500 c.f.u. ml^−1^, mixed in pairs at a 1:1 ratio (input) and injected in larvae as described above (groups of 5–10 larvae per mix). Each input ratio (strain A/strain B) was confirmed by plating an aliquot on PIA plates and replica plating at least 200 colonies on gentamicin-supplemented PIA plates. At 18 hpi, 10 µl of hemolymph was collected by pricking the abdomen of each larva with a sterile needle. Serial dilutions of the collected hemolymph (outputs) were plated on PIA and incubated overnight at 37 °C. The next day, at least 200 colonies from each larva were replica plated on gentamicin-supplemented PIA plates to evaluate the strain A/strain B ratio in the outputs. Each competitive index (CI) was calculated using the formula *CI=output (strain A/strain B)/input (strain A/strain B*). By this formula, CI>1 if strain A outcompetes strain B, CI<1 if strain B outcompetes strain A, and CI=1 if strain A/strain B has an equal ratio in the input and the output (same fitness of the two strains).

### *G. mellonella* luminescence analyses

At specific time intervals, the larvae injected with *P. aeruginosa* strains carrying P*zrmA*-lux, P*czcA*-lux or P*rpsL*-lux plasmids were transferred in a 12-microwell plate, and the luminescence, expressed as RLUs, was recorded by the SUNRISE microtiter-plate reader with an integration time of 1000 ms. The Chemidoc imaging system (Bio-Rad Laboratories) was used to take bright-field and chemiluminescent pictures of the larvae at representative time points after the injection, with an exposure time optimized to 120 s for the chemiluminescent setting mode.

### RNA extraction, reverse transcription and real-time quantitative PCR

*P. aeruginosa* RNA was extracted from infecting bacteria following a protocol already described [31], with minor modifications. Briefly, at 18 hpi, groups of five infected larvae were anesthetized on ice for 10 min before the tail was cut and the hemolymph recovered. Samples were treated with RNAprotect and extracted with the RNAeasy Kit (Qiagen), according to the manufacturer’s protocol, with the addition of DNase (Qiagen) and lysozyme (Sigma-Aldrich).

*G. mellonella* RNA was extracted from the hemolymph using the Total RNA Purification Kit (Norgen Biotek Corp.).

RNA concentration was determined with a NanoDrop™ Lite Spectrophotometer (Thermo Fisher Scientific). From each sample, 1 µg of RNA was reverse transcribed with the PrimeScript RT Reagent Kit and gDNA Eraser (Takara Bio Inc.). The primers used for real-time quantitative PCR (qPCR) were designed using Primer3 (Table S2) [34]. Real-time qPCR reactions were performed in triplicate in 10 µL reaction mixtures containing cDNA 50 ng (*P. aeruginosa*) or 25 ng (*G. mellonella*), primers 0.3 µm and 50% PowerUp SYBR Green Master Mix (Thermo Fisher Scientific). Amplifications were performed in a QuantStudio 3 real-time PCR system (Thermo Fisher Scientific) thermocycler with the following parameters: (i) initial denaturation at 95 °C for 4 min; (ii) 40 cycles of denaturation at 95 °C for 20 s, primer annealing at 60 °C for 30 s and extension at 72 °C for 30 s; and (iii) melting curve, from 50 to 90 °C (rate: 0.58 °C every 5 s). The mRNA fold induction was calculated using the ΔΔCt method [35] and normalized to the following housekeeping genes: *rpsL* for *P. aeruginosa* and *ubiquitin* (LOC113509582) for *G. mellonella*.

### Statistical analyses

Statistical analyses were performed using GraphPad Prism Software v.10.1.1. Student’s t-test and Mantel–Cox test were calculated as specified in the figure captions.

## Results

### *P. aeruginosa* PA14 – *G. mellonella* infection model

**Fig. 1.**
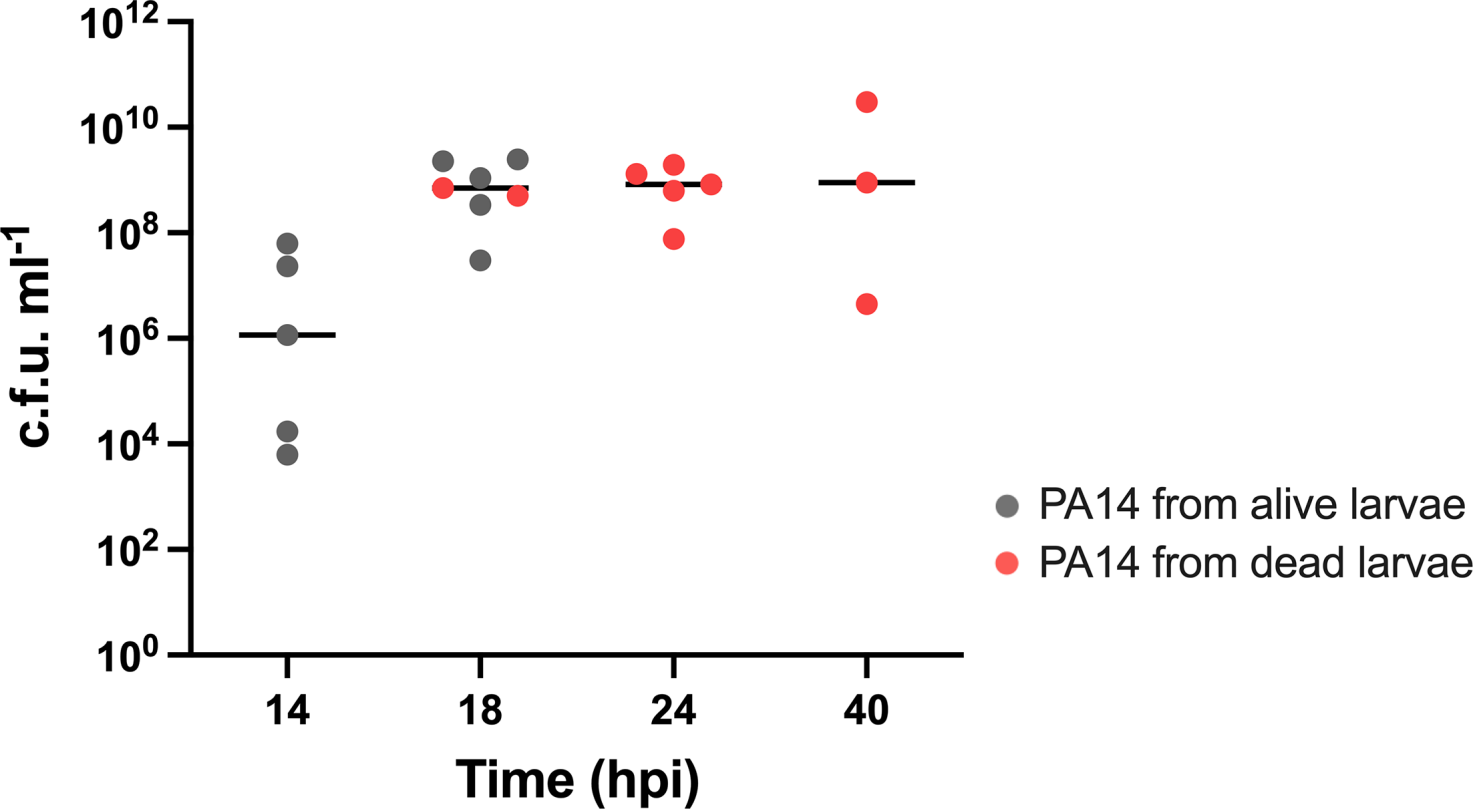
*P. aeruginosa* PA14 wt viability in *G. mellonella* larvae. An aliquot corresponding to 10 µl of the hemolymph of the infected larvae was collected, serially diluted and plated on PIA plates for c.f.u. counting. Results are expressed in c.f.u. ml^−1^ of hemolymph from alive (grey dots) and dead (red dots) larvae. The median value for each time point is represented with a horizontal line.

Preliminary experiments were performed to analyse the colonization of *P. aeruginosa* PA14 wt in the *G. mellonella* model. To this end, a group of 20 larvae was injected with PA14 wt strain (25 c.f.u. per larva). At different time points, the hemolymph from randomly selected larvae was collected and plated for viable bacteria counting. In all the experiments, only the hemolymph was considered for bacterial evaluation since a recent work demonstrated that *P. aeruginosa,* unlike other bacteria, localizes mainly within this compartment [36]. Fig. 1 shows the number of viable bacteria in infected larvae, demonstrating that the PA14 wt can rapidly colonize *G. mellonella*, consistent with previous studies [31,37]. The number of bacteria in the hemolymph increases over time, reaching a plateau at 18 hpi. Moreover, the larvae progressively showed symptoms of illness (loss of motility and melanization) until death, with no larvae surviving beyond 40 hpi. The 18 hpi time point was chosen for subsequent gene expression analyses and competition assays since the larvae were highly colonized, but most were still alive at this stage.

### *G. mellonella* transcriptional response to *P. aeruginosa* infection suggests the activation of Zn nutritional immunity mechanisms

**Fig. 2.**
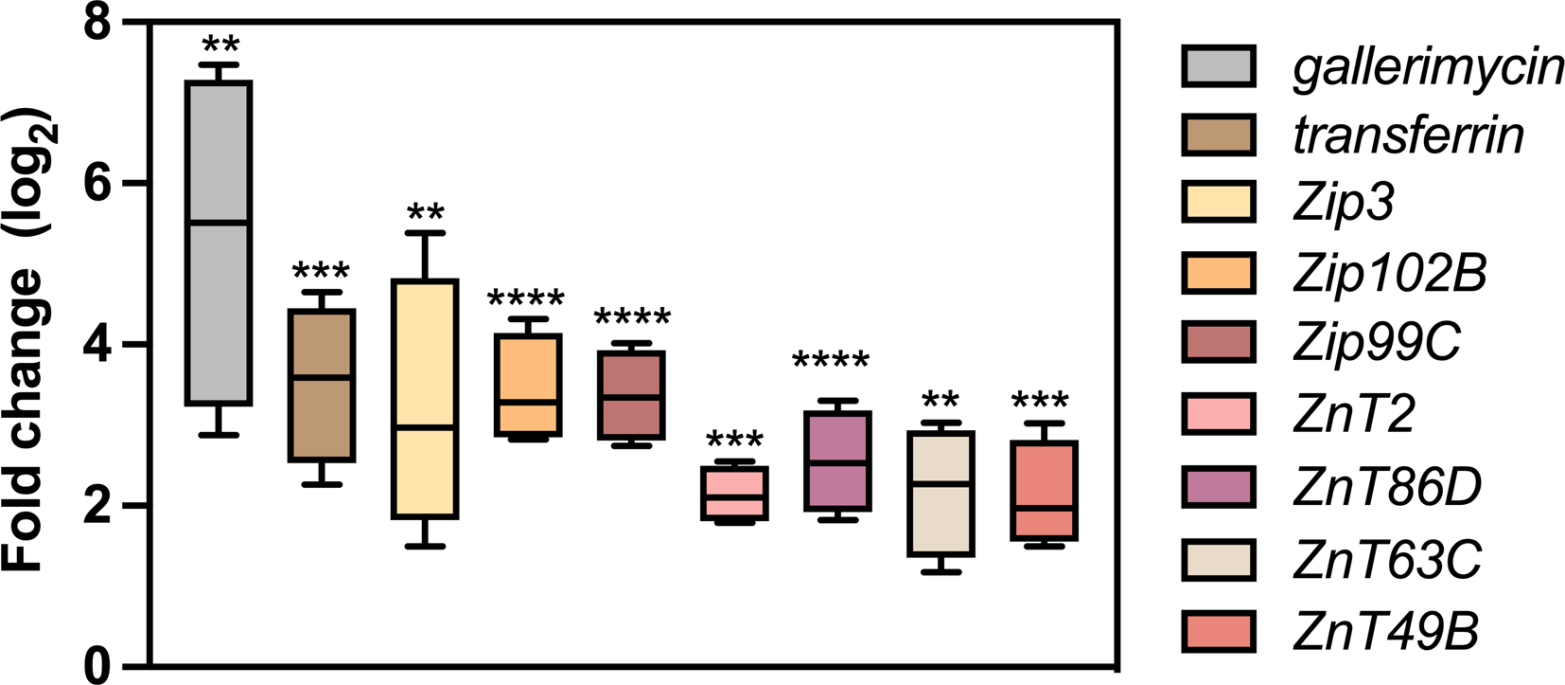
Expression of *G. mellonella* genes. Real-time qPCR was performed on *G. mellonella* mRNA extracted from infected larvae at 18 hpi. Data are graphed as a box plot, where the median value of three independent experiments, the interquartile range and the upper and lower values are represented for each data set. Statistical analyses were carried out using Student’s t-test. Asterisks indicate statistical differences in gene expression compared to PBS-injected *G. mellonella* (****P<0.0001; ***P<0.001; **P<0.01).

A survey in the National Center for Biotechnology Information (NCBI) genome data set (NCBI *Galleria mellonella* Annotation Release 101, GenBank assembly GCA_003640425.2), applying the filter ‘zinc’ to the gene name, retrieved 393 records. Among them, we selected seven records described as encoding Zn transmembrane transporter belonging to the ZIP family (ZRT, IRT-like protein) and the ZnT Cation Diffusion Facilitator (CDF) superfamily (Table 1) [38]. We analysed the expression of these genes in larvae infected with PA14 wt compared to the expression levels in PBS-injected larvae, along with the expression of genes known to be activated in response to pathogens, such as gallerimycin and transferrin [39,40]. As shown in Fig. 2, *G. mellonella* genes encoding for Zn transporters were significantly induced in PA14-infected larvae, as well as transferrin and the antimicrobial peptide gallerimycin. This suggests that similar to vertebrate hosts, *G. mellonella* mobilizes Zn to redistribute the metal within tissues and intracellular compartments to modulate metal availability during *P. aeruginosa* colonization.

**Table 1.** *G. mellonella* genes involved in Zn transport The correspondent gene IDs, a brief description and homologs are reported for each gene (from CSIRO_AGI_GalMel_v1).

Gene name	Gene description	Gene ID	Homolog
***Zip3***	Zinc transporter ZIP3-like; zinc ion transmembrane transporter activity	LOC113519639	*Drosophila melanogaster* Zip89B
***Zip102B***	Zinc/iron-regulated transporter-related protein 102B; zinc transporter ZIP9	LOC113514325	*Homo sapiens* SLC39A9
***Zip99C***	Zinc transporter Zip99C; zinc transporter ZIP13 homolog enables zinc ion transmembrane transporter activity	LOC113514493	*Homo sapiens* SLC39A13
***ZnT2***	Zinc transporter 2-like	LOC113519522	*Homo sapiens* SLC30A2
***ZnT86D***	zinc transporter 86D; enables zinc ion transmembrane transporter activity	LOC113519670	*Danio rerio* slc30a7
***ZnT63C***	Zinc transporter 63C; enables zinc ion transmembrane transporter activity	LOC113523582	*Danio rerio* slc30a1b
***ZnT49B***	Zinc transporter 49B; enables monoatomic cation transmembrane transporter activity	LOC113523578	*Homo sapiens* SLC30A9

### *P. aeruginosa* upregulates Zn uptake system in *G. mellonella* hemolymph

**Fig. 3.**
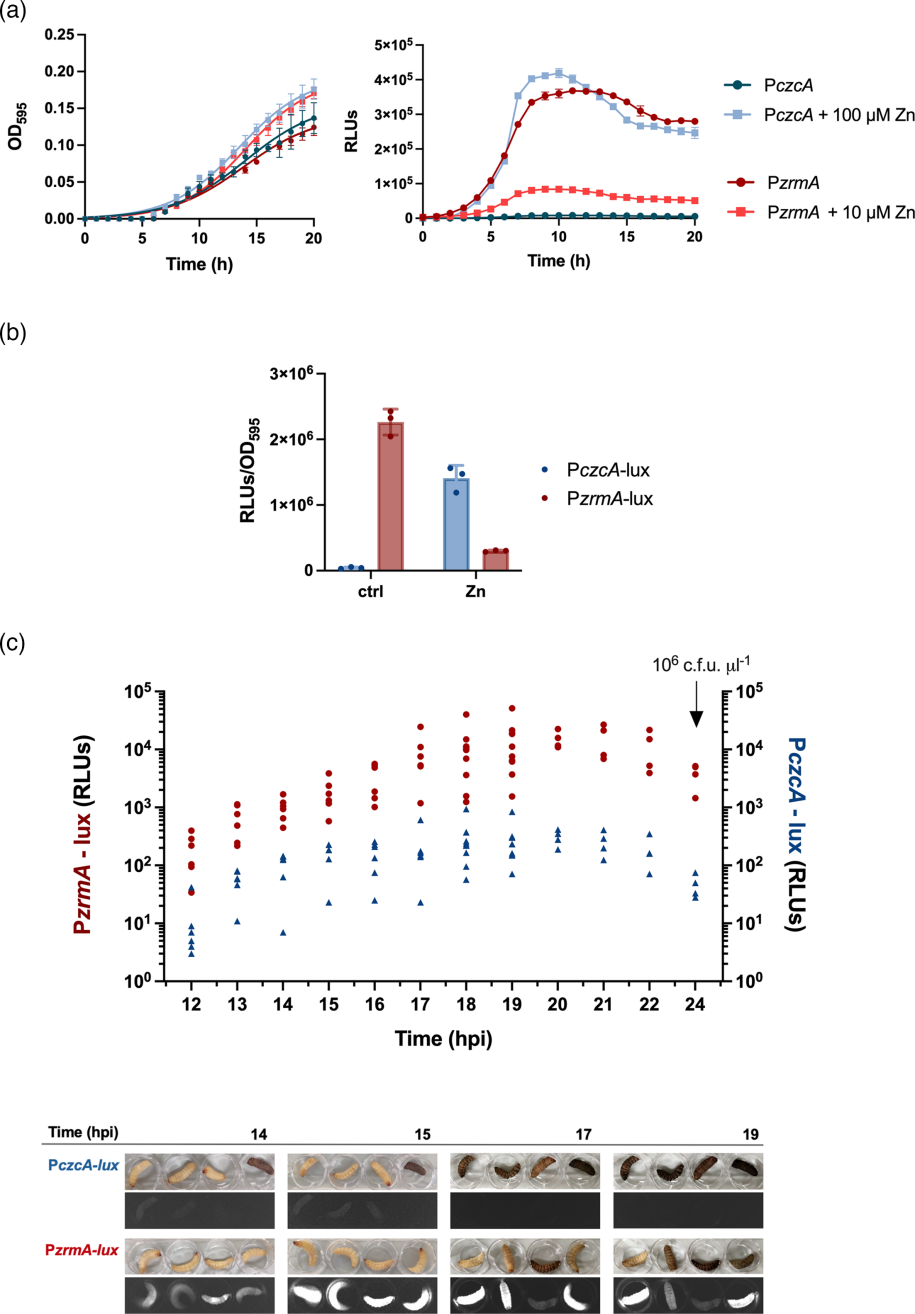
Analyses of *P. aeruginosa czcA* and *zrmA* promoter activity. (**a**) Growth curves (OD at 595 nm, OD_595_, left panel) and luminescence (RLUs, right panel) of PA14 wt strains carrying plasmids P*czcA*-lux and P*zrm*A-lux grown in E-VBMM supplemented or not with 10 or 100 µm ZnSO_4_. Each point indicates the mean value±sd of triplicates, and lines in the left panel represent nonlinear fit according to the Gompertz growth equation. (**b**) Representation of the endpoint (time=20 h) as RLUs per unit of OD_595_ (RLUs/OD_595_) of P*czcA*-lux and P*zrm*A-lux. (**c**) Luminescence (RLUs) of the infected larvae was measured every hour using the microplate reader. Each dot represents the luminescence from one larva, and the arrow indicates bacterial count from the hemolymph of the larvae at 24 hpi. In the bottom, images in the bright field and chemiluminescence settings showing four representative *G. mellonella* larvae infected with *P. aeruginosa* P*czcA*-lux or *P. aeruginosa* P*zrmA*-lux at 14, 15, 17 and 19 hpi.

The expression of two *P. aeruginosa* systems sensitive to Zn limitation and Zn excess, *zrmABCD* and *czcABC*, respectively, was monitored using a *lux* reporter plasmid carrying the *zrmA* and *czcA* promoter regions. PA14 wt strain was transformed with P*czcA*-lux or P*zrmA*-lux, and the responsiveness to Zn was first monitored during *in vitro* growth. The strains were inoculated in E-VBMM with or without different amounts of Zn, and both growth and luminescence were recorded over 20 h (Fig. 3a, b). A concentration of ZnSO_4_ 10 mmol l^−1^ was used to analyse the activity of the P*zrmA*-lux reporter, as it was previously demonstrated that this metal concentration reverses the phenotypes associated with Zn starvation [17]. In contrast, since the optimal Zn concentration for inducing *czcA* expression in E-VBMM is unknown, a preliminary screening with different Zn concentrations was conducted (Fig. S1). A 100 mmol l^−1^ ZnSO_4_ concentration was selected for further analyses, as it produced the highest P*czcA* induction without causing bacterial toxicity.

As expected, the activity of the *czcA* promoter was barely detectable in a Zn-depleted medium but increased upon Zn addition. Conversely, the *zrmA* promoter exhibited high activity under Zn-limited conditions, consistently decreasing upon Zn supplementation to the medium. Changes in luminescence were independent of the growth rate, as all the strains exhibited similar growth in the early exponential phase (8–12 h), while luminescence varied markedly. In later growth phases, when the presence of Zn similarly promoted the growth of PA14, the differences in promoter activities were even more pronounced.

The responsiveness of the reporter systems to Zn fluctuations prompted us to use the PA14 P*czcA*-lux and PA14 P*zrmA*-lux strains for injection experiments in *G. mellonella* to evaluate the activity of both promoters during the interaction with this model host. The luminescence in the infected larvae was monitored from 12 to 24 hpi (Fig. 3c), imaging and recording the RLUs every hour. As shown by the representative bright field images, larvae get progressively sick, turning black due to melanin accumulation [41], with no substantial changes relative to the injected bacterial strain. On the contrary, the luminescence pattern noticeably differs between the strains: the larvae colonized by PA14 P*czcA*-lux produced a faint signal compared to those injected with the P*zrmA*-lux. To better quantify differences in *luxCDABE* induction, the luminescence of the larvae was periodically recorded as RLUs (Fig. 3c, top). The induction of P*zrmA*-lux can be observed at 12 hpi with a median RLU value corresponding to ~2×10^2^ and exhibits a substantial increase over time, reaching a plateau at 18–21 hpi with luminescence values at ~10^4^ RLUs. In contrast, *czcA* promoter activity remained weak throughout the infection, with luminescence never exceeding 4×10^2^ RLUs, and showing a decreasing trend as the infection progressed.

At 24 hpi, the hemolymph from larvae was collected and plated for bacterial counts. No difference in the number of bacteria was observed (~10^6^ c.f.u. µl^−1^ of hemolymph in each larva), confirming that luminescence differences were due to promoter activity, not bacterial concentration inside the larvae. These results strongly suggest that *P. aeruginosa* faces Zn limitation rather than Zn excess during *G. mellonella* infection.

**Fig. 4.**
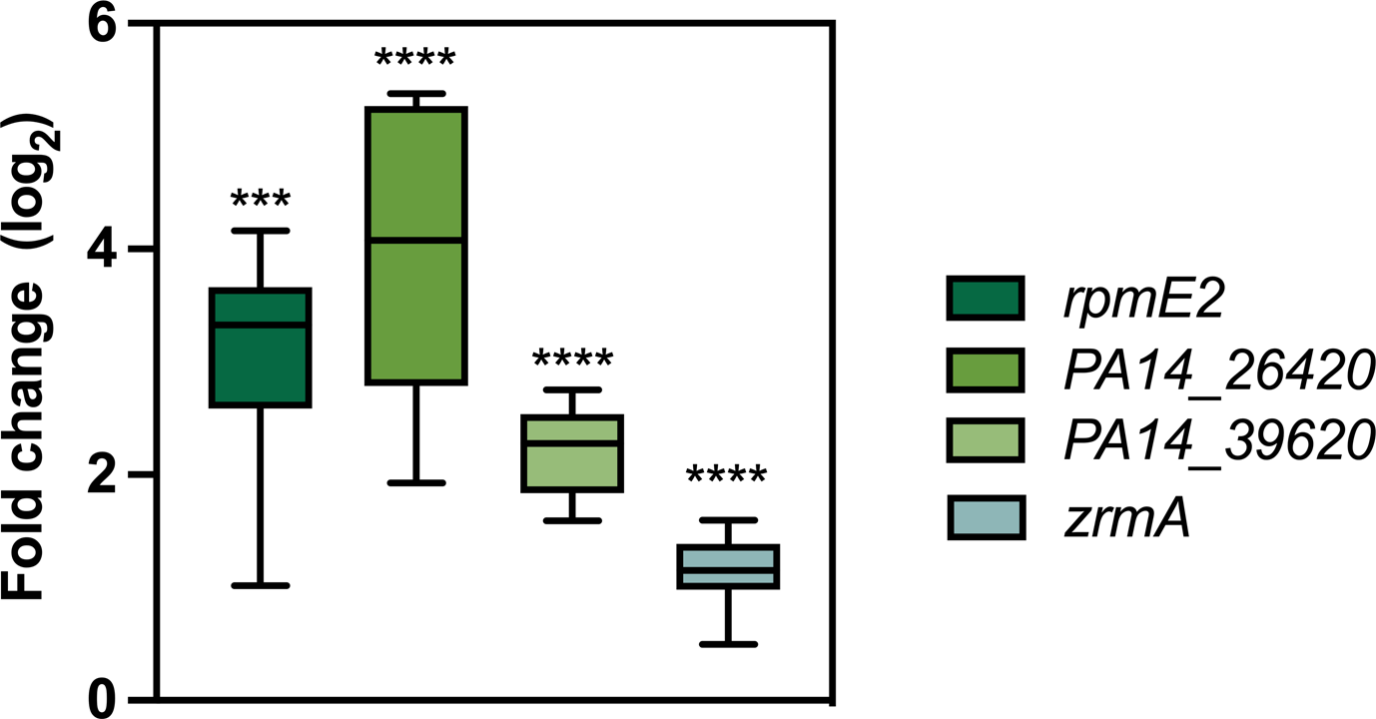
Expression of *P. aeruginosa* Zur-regulated genes from infected *G. mellonella* larvae. Real-time qPCR was performed on bacterial mRNA extracted from each infected larva at 18 hpi. Data are graphed as a box plot, where the median value of three independent experiments, the interquartile range and the upper and lower values are represented for each data set. Statistical analyses were carried out using Student’s t-test. Asterisks show statistical differences versus *P. aeruginosa* grown in LB medium (****P<0.0001; ***P<0.001).

To support this hypothesis, bacterial RNA was extracted from PA14 wt colonizing *G. mellonella*, and the expression of a group of Zur-regulated genes was analysed by real-time qPCR. Based on previous works, *rpmE2*, *PA14_26420* (*PA2911* in *P. aeruginosa* PAO1) and *PA14_39620* (*PA1925* in *P. aeruginosa* PAO1), together with *zrmA*, were chosen for the presence of a Zur-consensus sequence that elicits their upregulation in the transcriptional responses of *P. aeruginosa* PA14 to Zn restriction [17,42]. Consistent with the luminescence results, all genes exhibited significant induction at 18 hpi (Fig. 4), supporting the hypothesis that *P. aeruginosa* responds to Zn limitation during *G. mellonella* infection.

### *P. aeruginosa* Zn uptake systems are necessary for full virulence in *G. mellonella*

**Fig. 5.**
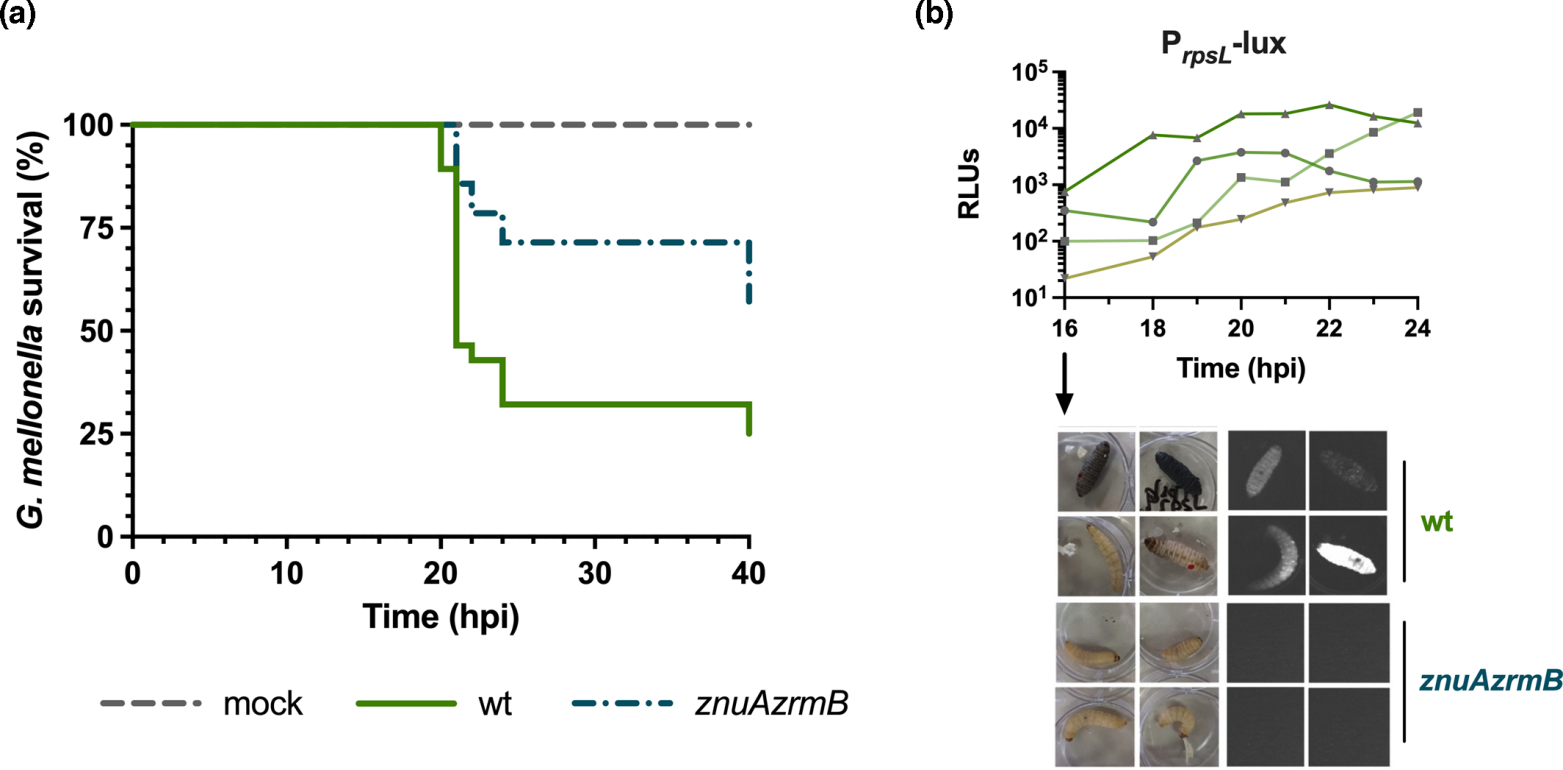
Contribution of *znuAzrmB* to *P. aeruginosa* colonization of *G. mellonella* (**a**) Kaplan–Meier survival curves of larvae infected with 25 c.f.u. per larva of the PA14 wt, the *znuAzrmB* mutant strain or PBS alone (mock). The percentage of surviving larvae over the total injected larvae (*n*=15) was monitored at different time points (hpi). Data have been pooled from three independent trials. Statistical analysis using the Mantel–Cox test indicates significant differences between the groups (*P=0.0084*). (**b**) The luminescence of four randomly chosen larvae infected with PA14 wt P*rpsL*-lux was measured using a microplate reader and reported in the graph as RLUs from 16 to 24 hpi (upper panel). Bright-field and chemiluminescence images of larvae infected with either PA14 wt P*rpsL*-lux (wt) or *znuAzrmB* P*rpsL*-lux at 16 hpi are shown in the lower panel. No luminescence is detected in larvae infected with the mutant strain.

To investigate whether *P. aeruginosa* high-affinity Zn uptake systems play a role in *G. mellonella* infection, groups of 15 larvae were inoculated either with the PA14 wt or the *znuAzrmB* mutant strain lacking components of the two primary Zn import systems, ZnuA and ZrmB. The PA14 wt exhibited high virulence, causing mortality in over 50% of the larvae within 20 hpi (Fig. 5, panel a). At later time points, ~25% of larvae survived the infection. In contrast, the virulence of the *znuAzrmB* mutant strain was significantly reduced, with the survival of 65% of larvae at 24 hpi. These data indicate that the proper functionality of *P. aeruginosa* Zn import systems is critical for full virulence in the *G. mellonella* model.

A comparison between PA14 wt and *znuAzrmB* mutant colonization of *G. mellonella* was performed following the luminescence of larvae infected with strains constitutively expressing the *luxCDABE* operon. The activity of the constitutive *rpsL* promoter cloned upstream to the *lux* operon was preliminarily tested *in vitro* and proved to be not responsive to Zn availability (Fig. S2).

*G. mellonella* larvae were then infected with PA14 wt or the *znuAzrmB* mutant strain, carrying the P*rpsL*-lux construct, and luminescence was monitored post-infection. As reported in Fig. 5b, the luminescence of the larvae infected with PA14 wt progressively increases from 16 hpi, with some variations among the different larvae. In contrast, the *znuAzrmB* mutant strain is undetectable at any time, indicating that the absence of Zn import systems impairs the ability of *P. aeruginosa* to replicate within *G. mellonella*. Moreover, all larvae infected with the mutant strain were alive at all time points, further supporting that bacterial Zn import systems affect the colonization ability and correlate with reduced virulence in the *G. mellonella* host.

**Fig. 6.**
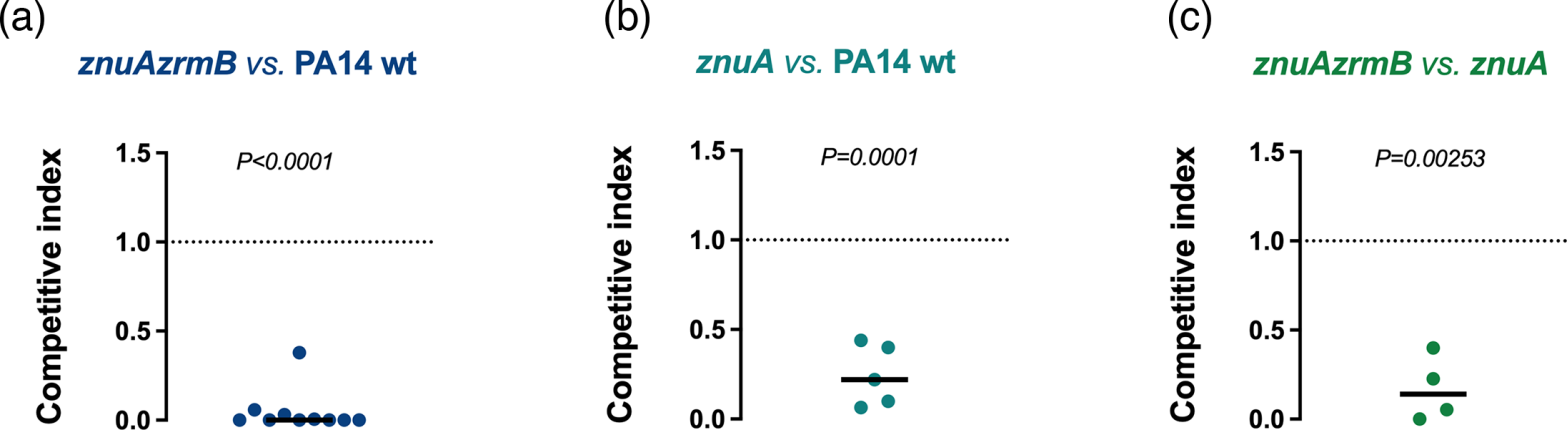
Competition assays in *G. mellonella*. (**a**) Competition between the *znuAzrmB* mutant (strain A) and the PA14 wt (strain B). (**b**) Competition between the *znuA* mutant (strain A) and the PA14 wt (strain B). (**c**) Competition between the *znuAzrmB* mutant (strain A) and the *znuA* mutant (strain B). In each case, larvae were injected with a 1:1 ratio of mixed bacterial inocula. Bacteria were recovered from the hemolymph of live larvae at 18 hpi, plated on PIA and replica plated on a selective medium to determine the output ratio. Each point represents the CI in an infected larva, and a horizontal straight line indicates the median values of the CI. Statistical analyses of the input and output ratios were performed using Student’s t-test.

A deeper analysis of the contribution of the ZrmABCD and ZnuABC Zn uptake systems was performed by competition experiments between pairs of *P. aeruginosa* strains for the colonization of *G. mellonella*. The larvae were injected with *P. aeruginosa* mixed inocula, and the competitive indexes of the strains were calculated at 18 hpi. As shown in Fig. 6, the *znuAzrmB* mutant strain is almost completely outcompeted by the PA14 wt strain (left panel), confirming the importance of both Zn uptake systems for the colonization of *G. mellonella*, as already suggested by the time-to-death experiment (Fig. 5). The competition between the *znuA* mutant and the PA14 wt strain (centre panel) demonstrates the critical role of Zn import via ZnuABC, as the wt strain outcompetes the mutant. Additionally, the *znuAzrmB* mutant is significantly outcompeted by the *znuA* strain (right panel), highlighting the importance of Zn import through pseudopaline. Together, these results provide evidence that both Zn import systems play a crucial role in the pathogenicity of *P. aeruginosa* during infection of *G. mellonella*.

### *G. mellonella* is a reliable platform for identifying genes involved in *P. aeruginosa* colonization

**Fig. 7.**
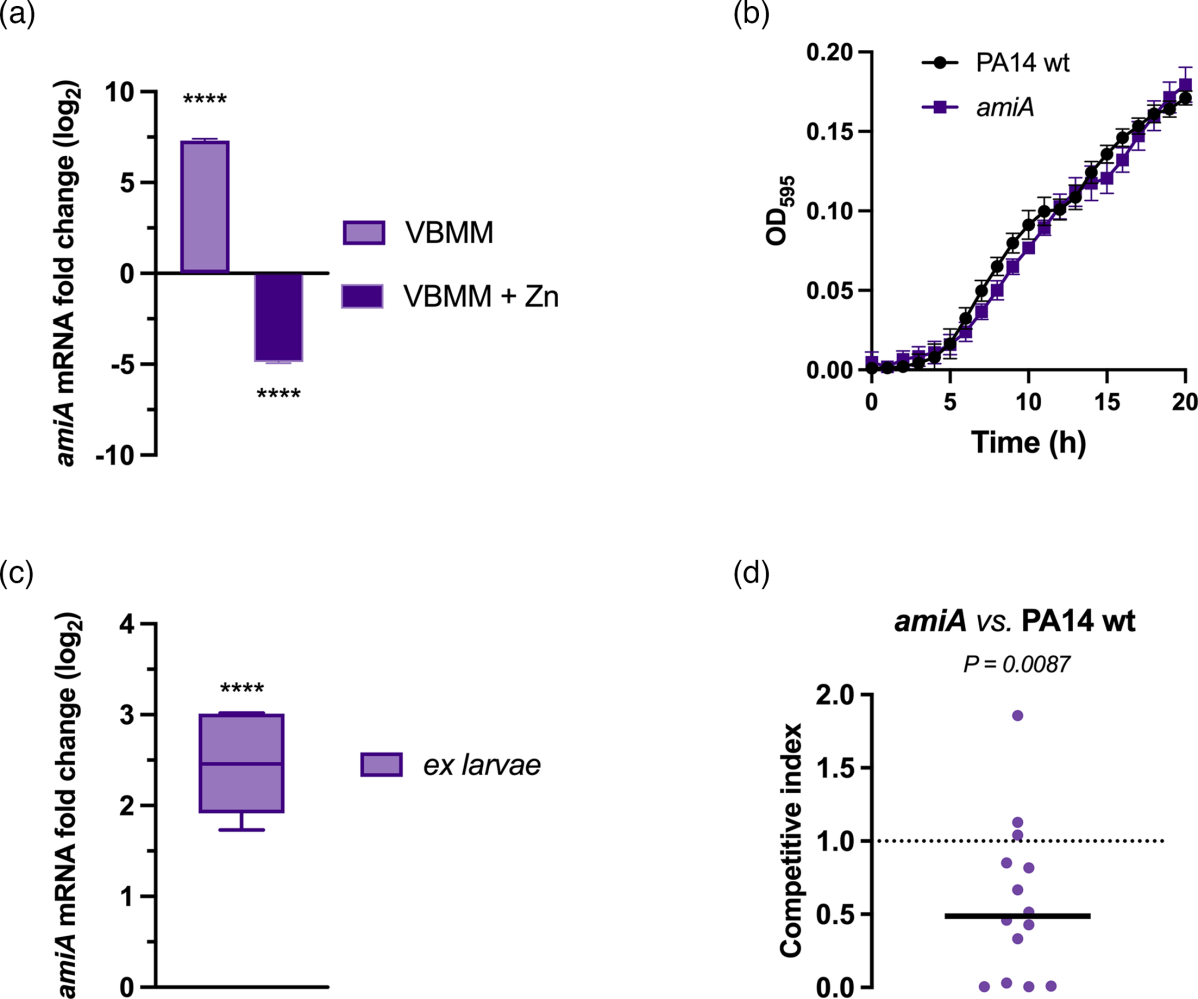
Regulation of *amiA* and its contribution to *P. aeruginosa* virulence. (**a**) The expression of *amiA* was analysed by real-time qPCR in the PA14 wt strain grown in VBMM compared to the PA14 wt grown in the LB medium (purple bar) and the PA14 wt grown in VBMM+ZnSO_4_ 2 µm compared to VBMM (dark purple bar). Statistical significances were calculated by Student’s t-test (****P<0.0001). (**b**) Growth curves of PA14 wt and *amiA* mutant strain in VBMM. (**c**) The expression of *amiA* was analysed by real-time qPCR in PA14 wt colonizing the hemolymph of infected larvae, compared to *amiA* expression in bacteria grown in LB medium. Statistical significance was calculated by Student’s t-test (****P<0.0001). (**d**) Competition assay in *G. mellonella* larvae injected with a 1:1 ratio of a mixed inoculum of *amiA* mutant strain (strain A) and PA14 wt (strain B). The bacterial output was recovered from the hemolymph at 18 hpi, plated on PIA plates and then replica plated on a selective medium for the output ratio counting. The CI in each larva were calculated as described in the ‘Methods’ section and reported as dots in the graph. The horizontal straight line indicates the median CI value from three independent experiments. Statistical significance was calculated using Student’s t-test.

Our results indicate that the response of *G. mellonella* to *P. aeruginosa* infection includes nutritional immunity strategies based on Zn sequestration. Therefore, we hypothesized that this model could be exploited for examining the role in virulence of novel *P. aeruginosa* genes associated with low Zn availability. One such gene, *amiA* (PA14_73040), has been identified as a component of the Zur regulon [17,42]. It encodes for an *N*-acetylmuramoyl-l-alanine amidase, a Zn-dependent exopeptidase involved in cell wall remodelling during bacterial cell division. *In vitro* gene expression analyses confirmed the responsiveness of *amiA* to Zn scarcity: the transcript is highly overexpressed in PA14 wt grown in a Zn-limiting medium compared to bacteria grown in LB medium, and its expression is repressed by Zn (Fig. 7a). However, despite its upregulation under Zn scarcity, we found that AmiA is not essential for *in vitro* growth, even in a Zn-restricted medium (Fig. 7b). The observed upregulation of *amiA* in Zn-limiting conditions led us to investigate whether it could have a role in infections. After injecting *G. mellonella* with the PA14 wt strain, we performed a gene expression analysis in bacteria recovered from the hemolymph at 18 hpi and observed a significant induction of *amiA* (Fig. 7c). To detect a possible contribution of *amiA* on *P. aeruginosa* host colonization, we performed competition assays in *G. mellonella* larvae using a mixed inoculum of PA14 wt and *amiA* mutant strain. As shown in Fig. 7d, even though we found some heterogeneity in the competition indexes, the median value indicates a significant disadvantage in colonization for the *amiA* mutant compared to the PA14 wt. These findings suggest that the *G. mellonella* infection mimics the nutritional immunity-driven microenvironment of the vertebrate host and could serve as a reliable platform for screening genes with an *in vivo* function that may not be evident *in vitro*.

## Discussion

Animal models are crucial in studying bacterial pathogenesis and developing treatments for infectious diseases. Research on *P. aeruginosa* pulmonary infections, particularly those associated with CF, has benefited from various murine models, including CF mice. However, these models only partially replicate the human CF condition [43]. A recent study has revealed significant differences between the *P. aeruginosa* transcriptome in CF sputum and those observed in acute lung infections in mice [44]. Larger mammals, such as pigs, more closely mimic the human response to *P. aeruginosa* infections, but their widespread use is limited by ethical and logistical challenges [45]. *In vitro* systems, such as CF human airway epithelial cells and synthetic CF sputum medium, have shown greater alignment with the *P. aeruginosa* transcriptome from human sputum [44]. Moreover, combining *in vitro* and *in vivo* approaches has been shown to more accurately capture *P. aeruginosa* gene expression and behaviour in chronic lung infections [46]. Nonetheless, these experimental setups are labour-intensive, costly and time-consuming. Alternative eukaryotic models offer several advantages, including ease of use, cost-effectiveness and relevance of the innate immune responses. Some of these models have proven valuable for initial investigations of novel pathogen virulence traits. For instance, zebrafish embryos (*Danio rerio*) are suitable alternative hosts for *P. aeruginosa*, and they are currently used in pre-clinical studies and early drug efficacy tests [26]. Recent research revealed that zebrafish replicate certain vertebrate innate immune responses, such as nutritional immunity mechanisms, with Zn transporters being upregulated in response to *P. aeruginosa* infection [27].

Over the past decade, *G. mellonella* has emerged as a suitable infection model for studying human pathogens*,* revealing that many virulence traits are also effective in this lepidopteran host [37,39, 47]. *G. mellonella* immune response shares notable similarities with the innate immune response of higher eukaryotes, featuring both humoral and cellular responses [48]. Its hemolymph, in analogy with the mammalian blood, is rich in immune cells capable of phagocytizing or encapsulating pathogens, while small antimicrobial proteins act as critical components of the humoral response [47]. Trace metals are essential in insect physiology and metabolism and play a role in response to pathogens [49,50]. Some studies have demonstrated the importance of Fe and Fe-binding proteins in the insect immune response since Fe levels affect the susceptibility to infections [50,51]. The melanization process, a major defence mechanism, is mediated by the Cu-dependent tyrosinase activity of phenoloxidases [52,54], and the peptidoglycan recognition proteins require Zn to act as modulators of immune signalling and as direct bactericidal molecules [50,55].

This study aimed to determine whether *G. mellonella* is a suitable model for studying the response of *P. aeruginosa* to host-imposed nutritional immunity strategies, particularly those involving Zn sequestration. *G. mellonella* possesses several Zn transporters from the ZIP/ZnT families, which share significant homology to those identified in other insects or vertebrates. However, their exact functions and localizations remain largely unknown. We observed that *P. aeruginosa* infection triggers the upregulation of ZIP and ZnT transporters, suggesting pathogen-induced Zn mobilization. ZIP3, whose homolog in *Drosophila melanogaster* (ZIP89B) is involved in dietary Zn assimilation by enterocytes [56], could reduce Zn levels in the hemolymph during infection since larvae were not fed during the experiment. The mammalian counterparts of the two other ZIP transporters analysed, SLC39A9 and SLC39A13, are likely involved in Zn homeostasis of the ER and trans-Golgi apparatus, probably loading Zn-containing proteins with the metal cofactor [57]. We can hypothesize that the upregulation of both these transporters in infected *G. mellonella* could be aimed at the secretion of Zn-dependent factors involved in the antimicrobial response. We also observed an increase in the mRNA levels of the ZnT transporters that, by homology with the vertebrate counterparts, could have a function in Zn eﬄux from the cytosol and in the compartmentalization of the cytosolic Zn into organelles [58]. It is worth mentioning that some ZnT transporters were already shown to be upregulated in zebrafish following *P. aeruginosa* colonization [27]. Consistent with broader nutritional immunity strategies, we also found increased expression of transferrin, a known mediator of Fe sequestration during infection [59].

The induction of a systemic remodelling of Zn distribution in *G. mellonella* as a response to pathogens colonization is supported by other recent findings. For example, during *G. mellonella* infection by the fungal human pathogen *Madurella mycetomatis*, a fungal zincophore was produced likely to counteract insect sequestration mechanisms [60]. At the same time, we cannot rule out the possibility that *G. mellonella* locally increases Zn concentration to intoxicate the pathogens (i.e. during phagocytosis by hemocytes) as suggested by the weak but detectable signal from the *PczcA*-lux reporter in infected larvae. Moreover, this low luminescence signal could have been partially quenched by the melanin, which may absorb emitted light. Host-induced Zn toxicity has been observed in other systems, such as elevated Zn levels to inhibit *Salmonella enterica* growth in plants [61], human macrophages trafficking Zn to phagosomes to poison intracellular pathogens like *Mycobacterium tuberculosis* and *S. enterica* [6,62] and the induction of *czcCBA* in *P. aeruginosa* during phagocytosis by macrophages in mice [21]. Recent studies in zebrafish embryos have suggested that *P. aeruginosa* can occupy distinct niches with varying Zn concentrations at different stages of infection, with some environments characterized by high Zn levels [27]. As a future goal, more detailed analyses of *P. aeruginosa* gene expression in both cell-free hemolymph and within hemocytes could provide valuable insights into Zn availability across different compartments of *G. mellonella*.

Our experiments also revealed strong activation of the *zrmA* promoter during colonization, and that other Zur-regulated genes were similarly induced. These include *rpmE2*, which encodes the ribosomal Zn-independent protein L31p that substitutes the Zn-containing paralog under Zn starvation [42]; PA14_26420 (PA2911 in *P. aeruginosa* PAO1), an importer of pyochelin bound to Zn or Co [14]; and PA14_39620 (PA1925 in *P. aeruginosa* PAO1), an uncharacterized protein induced in Zn-limited environments [17]. Notably, these genes are upregulated in *P. aeruginosa* grown in CF sputum, further supporting the relevance of Zn limitation during infection [63].

Time-to-death infection experiments highlighted the critical role of Zn import systems in *P. aeruginosa* virulence, as demonstrated by the significant reduction in bacterial virulence when both ZnuA and pseudopaline biosynthetic enzyme ZrmB were impaired. In line with this observation, the *in vivo* analysis through a bacterial constitutive luminescent reporter indicated that the burden of the *znuAzrmB* strain is not detectable in the larvae, which showed no symptoms. Furthermore, competition experiments reveal that *P. aeruginosa* relies on both Zn import systems to withstand Zn starvation imposed by the host during infection. Similar findings have been reported in fungi such as *Candida albicans* and *Cryptococcus gattii* [64,65] or in *Enterococcus faecalis* [66], where Zn import defects lead to reduced virulence. Likewise, reduced Zn import diminishes the ability of *Yersinia pestis* to colonize its natural insect vector, *Xenopsylla cheopis* [67].

To further investigate this concept, we explored the use of *G. mellonella* to identify genes involved in colonization within Zn-restricted host environments. The *P. aeruginosa* Zur regulon comprises many genes, some lacking a clear role in Zn starvation adaptation. Among them, *amiA* encodes for a Zn-dependent *N*-acetylmuramoyl-l-alanine amidase, involved in peptidoglycan cleavage during cell division. Several studies have established that amidases also play significant roles in pathogenesis. For instance, in *Helicobacter pylori*, amidase absence leads to unseparated cell chains, reduced motility despite functional flagella, increased amoxicillin tolerance and decreased colonization in the mouse stomach [68]. Similarly, in *Vibrio fischeri*, amidase mutations result in impaired motility and diminished host colonization [69]. Many species possess redundant peptidoglycan-modifying enzymes. *E. coli*, for instance, has three amidase paralogues that collectively regulate peptidoglycan cleavage [70]. *P. aeruginosa* produces two amidases, AmiA and AmiB, which are both Zn-dependent enzymes differing in the export pathway into the periplasm. AmiA, homolog to the *E. coli* AmiC, is exported by the Tat system, which requires that the substrate is folded and eventually loaded with the cofactor in the cytoplasm. In contrast, AmiB is exported by the Sec system, which exports unfolded substrates in the periplasm [71]. Since some studies have pointed out the importance of AmiB for cell division and proper maintenance of envelope permeability [72], little is known about the role and regulation of AmiA amidase.

Our data confirmed that *amiA* is induced by low Zn availability *in vitro*, as previously reported [42]. While *amiA* is not essential for growth *in vitro* under Zn limitation, we found it to be significantly upregulated during infection, and competition experiments showed that the *amiA* mutant was less successful at colonizing *G. mellonella* compared to PA14 wt. This finding confirms that bacteria recovered from the hemolymph of *G. mellonella* responded to Zn starvation and suggests that this infection model can reveal genes that are critical *in vivo* but might be overlooked *in vitro*, enhancing the utility of this model for studying host–pathogen interaction. An investigation of the precise role of the AmiA amidase at the host–pathogen interface is beyond the scope of this work and must be an object of future research. Little evidence of the involvement of Zur-regulated peptidoglycan-modifying enzymes in infection has been reported before. For instance, it was demonstrated that a Zn-regulated peptidase helps maintain cell wall integrity in *Acinetobacter baumannii* under conditions of immune-mediated nutrient sequestration, hypothesizing that it might modify the peptidoglycan to facilitate metal uptake [73]. We can speculate that *P. aeruginosa* has developed a similar mechanism, where the production of a Zur-regulated amidase supports essential cellular functions during severe Zn limitation, ensuring the proper placement of Zn transporters on the bacterial membrane. Another possibility is that since AmiA export is Tat-dependent, it is folded and loaded with Zn in the cytoplasm where Zn concentration must be ensured over a certain threshold. This would provide the proper activity of at least one Zn-dependent amidase, even in severe Zn-restricted conditions imposed by nutritional immunity during host colonization.

In conclusion, our findings highlight that *P. aeruginosa* experiences Zn limitation within the *G. mellonella* infection model. We demonstrate that the infected larvae actively mobilize Zn as a response to bacterial colonization and that *P. aeruginosa* must rely on its Zur-dependent Zn uptake systems to retain full virulence. The results suggest that *G. mellonella* effectively replicates critical aspects of nutritional immunity observed in higher organisms, making it a valuable model for studying the role of Zn in microbial pathogenesis. This system offers a robust platform for *in vivo* preliminary screening of genes potentially involved in pathogenicity, supporting its use for investigating host–pathogen interactions and Zn-based nutritional immunity.

## supplementary material

10.1099/mic.0.001524 Uncited Supplementary Material 1.

## Abbreviations

CF: cystic fibrosis
CI: competitive index
hpi: hours post-infection
LB: Luria–Bertani
PIA: *Pseudomonas* Isolation Agar
RLUs: relative luminescence units
Zur: Zn uptake regulator
