# Supplementary material for: Modelling host–pathogen interactions: Galleria mellonella as a platform to study Pseudomonas aeruginosa response to host-imposed zinc starvation
Source: Microbiology (Reading). 2025 Jan 22;171(1):001524. doi: 10.1099/mic.0.001524 (PMC11753293; doi:10.1099/mic.0.001524)
Supplement: Uncited Supplementary Material 1. [file mic-171-01524-s001.pdf]

Supplementary Material

Supplementary Tables

Table S1. Bacterial strains and plasmids.

***P.aeruginosa* PA14 strains**	**Relevant genotype**	**Reference/source**
wild-type		Lab collection
znuA	*znuA*::Gm	(13)
znuA::FLP	*znuA*::scar	(13)
znuAzrmB	*znuA*::scar *zrmB*::Gm	(14)
znuAzrmB::FLP	*znuA*::scar *zrmB*::scar	(14)
amiA	*amiA*::Gm	This work
***E. coli* strains**	**Relevant genotype**	**Source**
Hb101	F^-mcrBmrr *hsdS20*(r_B^- m_B^-) *rec*A13 *leu*B6 *ara*-14 *pro*A2 *lacY1 gal*K2 xyl-5 mtl-1	Lab collection
DH5α	φ80 Δ*lac*Z 15Δ (*lac*-*arg*F) U169 *deo*R*rec*A1 *end*A1 *hsd*R17 (rk^-, mk^+) *pho*A*sup*E44 λ^-*thi*-1	Lab collection
**Plasmids**	**Description**	**Reference/source**
pETSlux	Broad host range, promoterless *luxCDABE*; Gm^R	(31)
P*zrmA*-lux	pETSlux carrying *zrmA* promoter; Gm^R	This work
P*czcA*-lux	pETSlux carrying *czcA* promoter; Gm^R	This work
P*rpsL*-lux	pETSlux carrying *rpsL* promoter; Gm^R	This work
pEX18Tc	Broad-host-range gene replacement; $sacB^+$;Tc^R,$oriT^+$	(32)
pPS856	Source of gentamicin resistance cassette. Amp^R, Gm^R	(32)
pRK2013	Broad-host-range helper vector; Kan^R	Lab collection

Table S2. Primers used in this work. All primers were purchased from Sigma-Aldrich.

**Target**	**Forward (5′-3′)**	**Reverse (5′-3′)**	**Source or ref**
***P. aeruginosa* PA14 promoter-lux cloning**			
zrmA	ATGAGCTCGCGCCAGGGTGCGGCTGA	GGGAATTCGGGAAATCGCACCAGAAAAGAA	This work
czcA	ATGAGCTCGTTTCATCCAACTATTGCCA	GCGAATTCGTTCCGCTCCTCGTCTGC	This work
rpsL	ATGAGCTCAAGCCGCAGCGTGTGAGT	ATGAATTCCTATAGCTCCACTGATTGTC	This work
***P. aeruginosa* PA14 mutant construction**			
5’ amiA	ATAGAATTCCGAAGTCCTGGTCGAAGC	ATTAGGATCCGAGCAGGCTTTGCAGGAG	This work
3’ amiA	ATAGGATCCGTTCTCGGCCAGTTGTCG	ATTAAGCTTCTCCACCTTTACTGCCTG	This work
check amiA	CTCGCCCTGTTCCAGTTC	CAGACCAATACGCGCTAC	This work
***P. aeruginosa* PA14 RT-qPCR**			
PA14_39620	ACCTGATCGACCTGTTCCTG	GGATTCTCGACCACCACCA	This work
PA14_26420	CTACATCGACCCCTGGCATC	GCGGTCGATATGGTTCTGGT	(14)
rpmE2	GCCGACGTGTACTTCCTGAT	GCGTCACGTAGGGATAGGTC	(27)
zrmA	GACACCCGTATCGAGGACAT	GAAGCCACGGACGTTGTACT	(27)
amiA	GCTACAACGCCGACATGTTC	GGAGAGGGCATATACCGATG	This work
rpsL	GCTGCAAAACTGCCCGCAACG	ACCCGAGGTGTCCAGCGAACC	This work
***G. mellonella* RT-qPCR**			
gallerimycin	GAAGTCTACAGAATCACACGA	ATCGAAGACATTGACATCCA	(36)

transferrin	AAACAGTCCTGCATTGTCGG	CATTTGTCTGGGTGTTGGCA	This work
Zip3	TTTGTTGCCGGAAGTGGATG	CATGAAGAAGCCAGCACACA	This work
Zip102B	TACTGTGGGTCTGGTGGTTC	GGCGCCTTGTGTAGCATAAT	This work
Zip99C	GGAGGACGATGAACACAAGC	TACCCCGTTGTTTCCTCTCC	This work
ZnT2	GTGCTTGGTGACTTCTTGCA	GAAGAGGAACGTGCAGATCG	This work
ZnT86D	ATGTTCTTCGATTGCACGGG	AAAGCCTGCCAACACTTCAG	This work
ZnT63C	TCAGCATAACAGTGGAGGCT	ATCCCACAACGTTAAGCAGC	This work
ZnT49B	TATTTTGTCCTTGGCGGTGC	TGCGCATAACATACTCGTGC	This work
ubiquitin	TCAATGCAAGTAGTCCGGTTC	CCAGTCTGCTGCTGATAAACC	(37)

Supplementary Figures

Figure S1

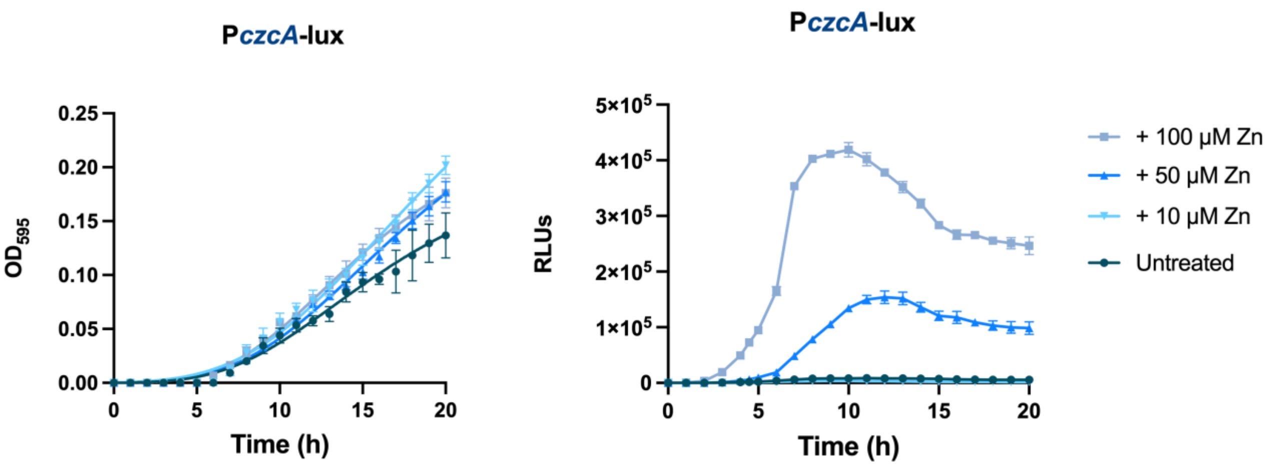

Dose-response to Zn of the P*czc*A-lux reporter strain. Growth (left panel) and luminescence (right panel) of PA14 carrying the reporter plasmid P*czc*A-lux in E-VBMM supplemented or not with an increasing amount of $ZnSO_4$, as indicated in the legend.

Figure S2.

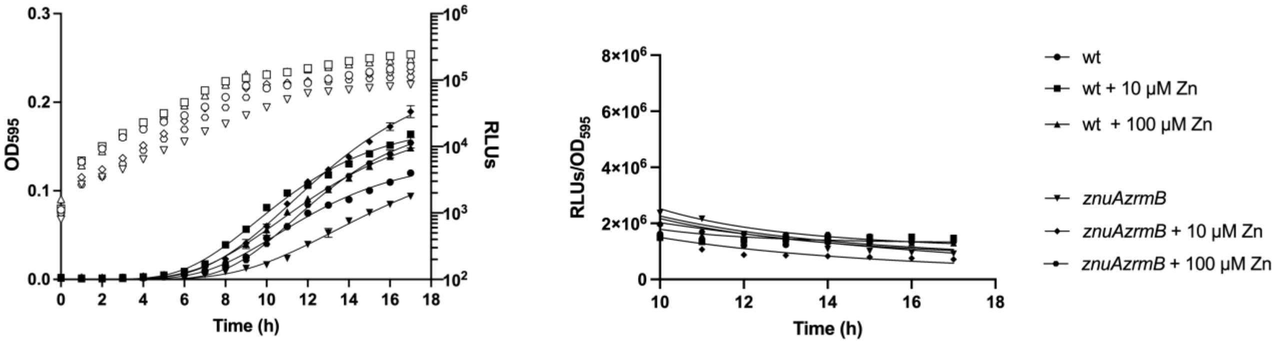

***In vitro* analyses of *rpsL* promoter activity in response to Zn availability.** PA14 wild-type and the *znuAzrmB* mutant strain carrying plasmid P*rpsL*-lux were grown in E-VBMM supplemented or not with 10 or 100 µM $ZnSO_4$. In the left panel, Luminescence (RLUs, empty symbols) and optical densities (OD_{595}, filled symbols) were recorded every hour and plotted. Each point indicates the mean value ± SD of triplicates, and lines represent nonlinear fit according to the Gompertz Growth equation. The right panel shows the RLUs corresponding to the exponential growth phase, normalized per OD units, showing that the luminescence depends only on the bacterial OD_{595} and not on the presence of Zn itself.
